# Histone Deacetylases Cooperate with NF-κB to Support the Immediate Migratory Response after Zebrafish Pronephros Injury

**DOI:** 10.3390/ijms23179582

**Published:** 2022-08-24

**Authors:** Mingyue Zhuang, Alexander Scholz, Gerd Walz, Toma Antonov Yakulov

**Affiliations:** 1 Renal Division, University Freiburg Medical Center, Faculty of Medicine, University of Freiburg, Hugstetter Strasse 55, 79106 Freiburg, Germany; 2 Signaling Research Centres BIOSS and CIBSS, University of Freiburg, Albertstrasse 19, 79104 Freiburg, Germany

**Keywords:** acute kidney injury, HDAC, histone deacetylase, zebrafish pronephros, directed cell migration, laser ablation, epigenetic modifiers

## Abstract

Acute kidney injury (AKI) is commonly associated with severe human diseases, and often worsens the outcome in hospitalized patients. The mammalian kidney has the ability to recover spontaneously from AKI; however, little progress has been made in the development of supportive treatments. Increasing evidence suggest that histone deacetylases (HDAC) and NF-κB promote the pathogenesis of AKI, and inhibition of Hdac activity has a protective effect in murine models of AKI. However, the role of HDAC at the early stages of recovery is unknown. We used the zebrafish pronephros model to study the role of epigenetic modifiers in the immediate repair response after injury to the tubular epithelium. Using specific inhibitors, we found that the histone deacetylase Hdac2, Hdac6, and Hdac8 activities are required for the repair via collective cell migration. We found that *hdac6*, *hdac8*, and *nfkbiaa* expression levels were upregulated in the repairing epithelial cells shortly after injury. Depletion of *hdac6*, *hdac8*, or *nfkbiaa* with morpholino oligonucleotides impaired the repair process, whereas the combined depletion of all three genes synergistically suppressed the recovery process. Furthermore, time-lapse video microscopy revealed that the lamellipodia and filopodia formation in the flanking cells was strongly reduced in *hdac6*-depleted embryos. Our findings suggest that Hdac activity and NF-κB are synergistically required for the immediate repair response in the zebrafish pronephros model of AKI, and the timing of HDAC inhibition might be important in developing supportive protocols in the human disease.

## 1. Introduction

Acute kidney injury (AKI) is a serious disease complication in hospitalized patients and accounts for about 13.3 million cases and up to 2 million deaths globally each year [1,2]. The high morbidity, mortality, and high medical costs have made AKI a global concern and a central research topic over the last few decades. The human kidney possesses the remarkable ability to recover from tubular epithelium injury. However, despite the developments in supportive care, the recovery process remains unpredictable and often incomplete in nature.

Cell labeling studies in the murine model have demonstrated that in the mammalian kidney, tubular epithelium is repaired by surviving epithelial cells, and this process does not require specialized progenitors [3,4,5]. It appears that resident epithelial cells de-differentiate and proliferate to replace the damaged, shed-off cells. However, the processes governing the early events after tubular injury remain unknown, mainly due to technical limitations, and the limited accessibility of the murine kidney. Only recently has it become possible to visualize the early repair events in a zebrafish model of AKI [6,7]. The transparent, easily accessible by time-lapse microscopy zebrafish embryo features a fully functional embryonic kidney, the pronephros. In this process, 2-photon laser-mediated injuries to the pronephros are rapidly repaired via directed cell migration [6,7]. The surviving tubular epithelium cells flanking the injury migrate towards each other to close the gap and re-establish the patency of the duct. This process is characterized by the significant increase in migration speed, and a reversal in the direction of migration of the proximal epithelium [6,7]. It is accompanied by changes in gene expression, and depends on the Myc transcription factor, as well as on the Cxcr4–Cxcl12 signaling axis [7,8]. The increase in migration speed during repair is powered through a metabolic switch from oxidative phosphorylation to glycolysis, whereas purinergic signaling in the surviving epithelial cells appears to play a role in the directionality of migration [7,8].

Many of the transcriptional changes in the kidney epithelium after AKI can be traced back to epigenetic regulation of gene expression [9,10,11]. Histone modifications such as histone methylation and histone acetylation have become recently focused on in research for their role in AKI. Methylation of histone lysine or arginine residues is regulated by methyltransferases and demethylases. Histone methylation orchestrates transcription via the generation of docking sites for chromatin modifiers, and can result in active, poised, or repressive status of chromatin marks [12]. From the lysine-specific methyltransferases, inhibition of EZH2 with 3-deazaneplanocin A (3-DZNeP) ameliorates the disease progression in a mouse ischemia/reperfusion model [13]. Furthermore, the EZH2 inhibitor zld1039 reduced inflammation in cisplatin-induced AKI [14]. Proposed mechanisms for the beneficial effect of EZH2 inhibition in AKI include suppression of the NF-κB p65 signaling [14], and modulation of the JNK/FoxO3a pathway [15].

Histone acetylation requires histone acetyl transferases (HAT) that transfer an acetyl group to lysines. This process is generally associated with active promoters. In contrast, histone deacetylases (HDAC) remove acetyl groups, which causes chromatin condensation, and repression of gene expression [9,10,11]. HDAC have received special attention in recent years, because of their regulation in murine AKI models [16,17,18,19,20,21,22]. HDAC have been grouped into four classes: class I (HDAC1, 2, 3, and 8); class II (HDAC4, 5, 6, 7, 9, and 10); class III (SIRT1–7); class IV (HDAC11). Class II HDAC has been further sub-divided into class IIa (HDAC4, 5, 7, and 9) and class IIb (HDAC6 and 10) [23]. The role of class I HDAC in AKI is controversial: blocking class I HDAC activity with MS-275 resulted in a more severe tubular injury in mouse models of AKI induced by folic acid or rhabdomyolysis [16], whereas HDAC inhibition with phenylthiobutanoic acids enhanced renal recovery in a mouse ischemia/reperfusion model [24]. Inhibition of class II HDAC appears to have a renoprotective effect in murine models of AKI. Several studies have demonstrated a renoprotective function for HDAC6 inhibition. Blocking HDAC6 with tubostation A or with compound 23BB significantly improved renal function and alleviated renal tubular injury in AKI models induced by rhabdomyolysis or cisplatin [18,20,21,25,26]. HDAC5 inhibition leads to increased histone acetylation and BMP-7 expression, which in turn might enhance post-ischemic regeneration by directly antagonizing the Smad signaling pathway [27,28,29]. Most of the aforementioned studies were conducted in mouse models and report on later processes, such as cell proliferation and renal fibrosis. However, the functions of histone modifiers in the early migratory response remain unclear. Here, we found that HDAC activity and NF-κB signaling cooperate to support the immediate repair response after tubular injury.

## 2. Results

### 2.1. Histone Deacetylase (Hdac) Activity Is Necessary for Pronephros Repair after Laser-Induced Injury

To examine the role of histone modifying enzymes in the pronephros repair, we used a 2-photon laser to cause tubular injuries in pharmacologically treated 2-day-old zebrafish. This induces an immediate migratory response in the flanking epithelium, which closes the gap within 24 h post injury (hpi) [7,8]. Embryos were incubated in inhibitors of histone modifiers for one hour before injury and for 24 hpi, and the repair success was quantified using fluorescent microscopy.

The primary function of EZH2 as a part of the PRC2 complex (Polycomb Repressive Complex 2) is the trimethylation of H3K27, which is a mark for transcriptional repression [30]. In contrast, JMJD3 and its paralog UTX remove methyl groups from the H3K27me3, thereby activating transcription at target promoters [31,32]. Treatment with increasing concentrations of GSK-J4, a potent dual inhibitor of Jmjd3/Kdm6B or Utx/Kdm6A (IC50 of 60 nM) did not affect the repair process (Figure S1A). Similarly, embryos treated with the Ezh2 inhibitor CPI-169 (IC50 of 0.24 nM) repaired normally (Figure S1B). Thus, the methylation status of Lys-27 on histone 3 (H3K27) does not affect the pronephros repair after injury.

DOT1L is a histone 3 Lys-79 methyltransferase that catalyzes the mono-, di-, and trimethylation of H3K79, and is generally associated with active transcription [33,34]. Inhibition of Dot1l with the specific inhibitor Pinometostat (EPZ5676) did not delay the repair process in 2-day-old zebrafish embryos, suggesting that the Dot1l activity is not required for the repair process (Figure S1C).

The MLL1 enzyme catalyzes mono-, di- and trimethylation of H3K4, a mark of active transcription [35]. In contrast, the lysine-specific histone demethylase 1 (LSD1) removes monomethyl and dimethyl groups from lysine 4 in histone H3 (H3K4me1/2) [36,37]. We found that embryos treated with the Mll1 inhibitor MM-102 (IC50 of 0.4 μM) repaired the pronephros normally after injury (Figure S1D). Similarly, embryo treatment with the specific Lsd1 inhibitor RN-1 (IC50 of 70 nM) had no effect on the repair process (Figure S1E). Thus, our results indicate that the Lsd1 and the Mll1 activities are dispensable for the migratory response.

**Figure 1:**
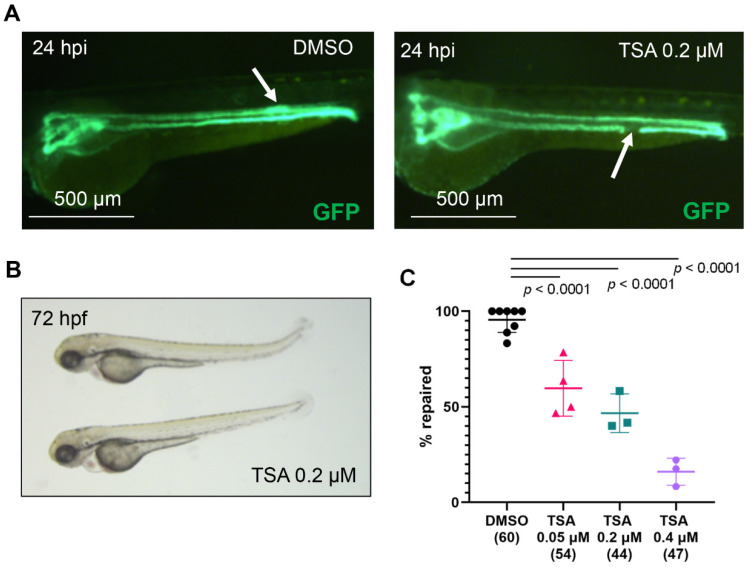
Histone deacetylases are required for zebrafish pronephros repair after injury. (**A**) The pronephros in a control, DMSO-treated embryo of the transgenic *Tg(wt1b:GFP; cdh17:GFP)* zebrafish line is fully repaired at 24 h post injury (hpi, left panel). The white arrow points to the location of the repaired tubule, which is characterized by a typical thickening of the duct. In contrast, a TSA-treated embryo fails to repair the pronephros (right panel). The arrow points to the ablated portion of the tubule. (**B**) Zebrafish embryos, incubated in 0.2 µM TSA for one hour before ablation and 24 h post ablation developed normally. (**C**) TSA treatment significantly reduced the number of embryos that repaired the tubule after laser-induced injury in a concentration-dependent manner. The concentration (micromolar, µM) is shown below each group. The number of examined embryos is displayed in brackets. Individual experiments are plotted, and the mean and standard deviation for each group are displayed. Significance was calculated using Fisher’s exact test (two-tailed).

Chromatin deacetylation, carried out by histone deacetylases (HDAC), is generally associated with transcriptional silencing [23,38]. Embryos, treated with pan-Hdac inhibitor trichostatin A (TSA, IC50 of ~1.8 nM) developed normally (Figure 1 and Figure S2). Importantly, inhibition of Hdac activity with TSA significantly reduced the percentage of repaired pronephri in a concentration-dependent manner (Figure 1). Our result suggest that Hdac activity is necessary for the immediate repair response of the pronephric tubular epithelium after injury.

### 2.2. Role of Class I Hdac in the Zebrafish Pronephros Repair

Since there are several classes of HDAC, we wondered which HDAC could explain the effect of the pan-HDAC inhibitor TSA. Class I HDAC (HDAC1, HDAC2, HDAC3, and HDAC8) belong to the arginase/deacetylase superfamily and have similar sequences to the yeast Rpd3 protein [23]. Entinostat (MS-275) is a small molecule inhibitor of Hdac1 (IC50 of 0.51 μM) and Hdac3 (IC50 of 1.7 μM, Figure S3). Entinostat was shown to be nephroprotective and effectively ameliorated histological alterations in murine models of AKI [17,39]. Treatment of 2-day-old zebrafish for 24 h with 10 µM or 20 µM entinostat solution did not affect the embryonic development (Figure S4). Furthermore, entinostat-treated embryos repaired normally after injury (Figure S4). Thus, Hdac1 or Hdac3 activities do not affect the migration-based repair process.

**Figure 2:**
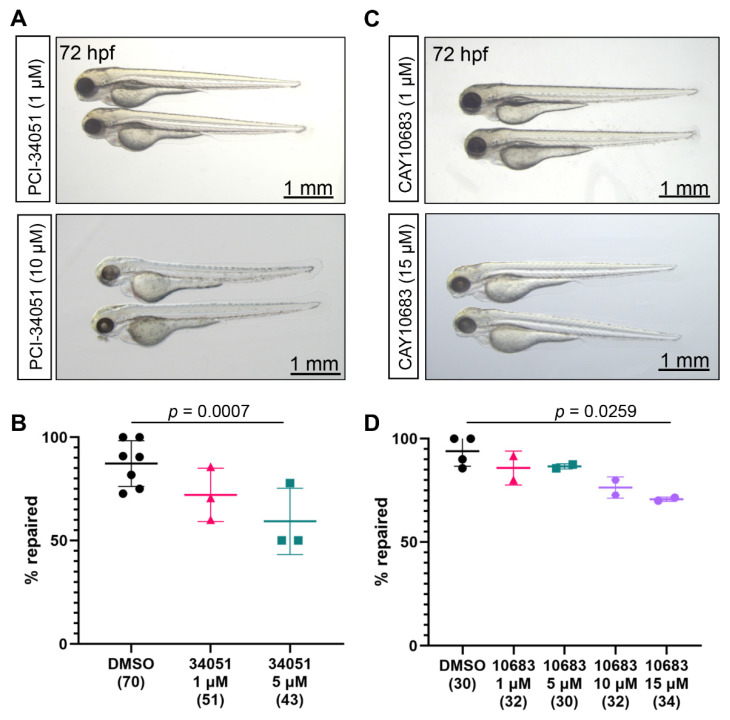
Hdac8 and Hdac2 activities are required for pronephros repair after laser-induced injury. (**A**) Embryos, incubated in 1 µM PCI-34051 (upper panel) or 10 µM PCI-34051 (lower panel) from 48 to 72 hpf developed normally. (**B**) PCI-34051 affects the pronephros repair in a concentration-dependent manner. Embryos were incubated in DMSO or increasing concentrations of the specific Hdac8 inhibitor PCI-34051 for one hour before ablation and for 24 h post injury (hpi). (**C**) Treatment of zebrafish embryos from 48 to 72 hpf with 1 µM or 15 µM solution of the Hdac2 inhibitor CAY10683 did not affect development. (**D**) Treatment of zebrafish larvae with 15 µM solution of CAY10683 (10683) significantly increased the percentage of embryos that did not repair the gap after 24 h. The numbers of examined embryos are displayed in brackets. The PCI-34051 concentrations (micromolar, µM) are shown below each group. The points represent individual experiments. Mean and standard deviation for each group are displayed. Significance was calculated using Fisher’s exact test (two-tailed).

The role of Hdac8 in kidney injury models is contradictory. On the one hand, Hdac8 overexpression has been shown to protect proximal tubular epithelial cells in hypoxia/reoxygenation-induced mitochondrial fission [40]. On the other hand, the potent Hdac8 inhibitor PCI-34051 (IC50 of 10 nM, Figure S3) was nephroprotective in a rodent model of AKI [19]. Incubation of zebrafish embryos in up to 10 µM PCI-34051 solution between 48 hpf and 72 hpf did not affect normal development (Figure 2A). Interestingly, PCI-34051 treatment significantly decreased the number of successful pronephros repairs in a concentration-dependent manner (Figure 2B). Our data indicate that Hdac8 activity is necessary for the tubular repair after laser-induced injury.

Next, we used the potent and selective Hdac2 inhibitor santacruzamate A (CAY10683, IC50 of 119 pM, Figure S3) to study the role of Hdac2 in pronephros repair. Incubation of zebrafish embryos in santacruzamate A solution for one hour before injury and 24 hpi had no toxic effects (Figure 2C). Importantly, higher concentrations of santacruzamate A significantly reduced the number of embryos that successfully repaired the pronephric tubules (Figure 2D), indicating that Hdac2 activity might be required for the pronephros repair.

### 2.3. Role of Class IIa and Class IIb Hdac in the Zebrafish Pronephros Repair

The class IIa (HDAC4, HDAC5, HDAC7, and HDAC9) and class IIb (HDAC6 and HDAC10) HDAC are similar in sequence to the yeast Hda1 protein [23]. To study the role of class IIa Hdac in the early tubular repair after injury, we used the potent class IIa Hdac inhibitors TMP269 and LMK-235 (Figure S3). TMP269 inhibits Hdac4, Hdac5, Hdac7, and Hdac9 with IC50 of 157 nM, 97 nM, 43 nM, and 23 nM, respectively. LMK-235 selectively inhibits Hdac4 (IC50 of 11.9 nM) and Hdac5 (IC50 of 4.2 nM). Two-day-old zebrafish embryos tolerated well up to 15 µM solution of TMP269 and up to 20 µM solution of LMK-235 for 25 h (Figure S5). However, neither TMP269 nor LMK-235 had an effect on the pronephros repair after injury (Figure S5), suggesting that class IIa Hdac activity does not play a role in the early migratory response after injury.

**Figure 3:**
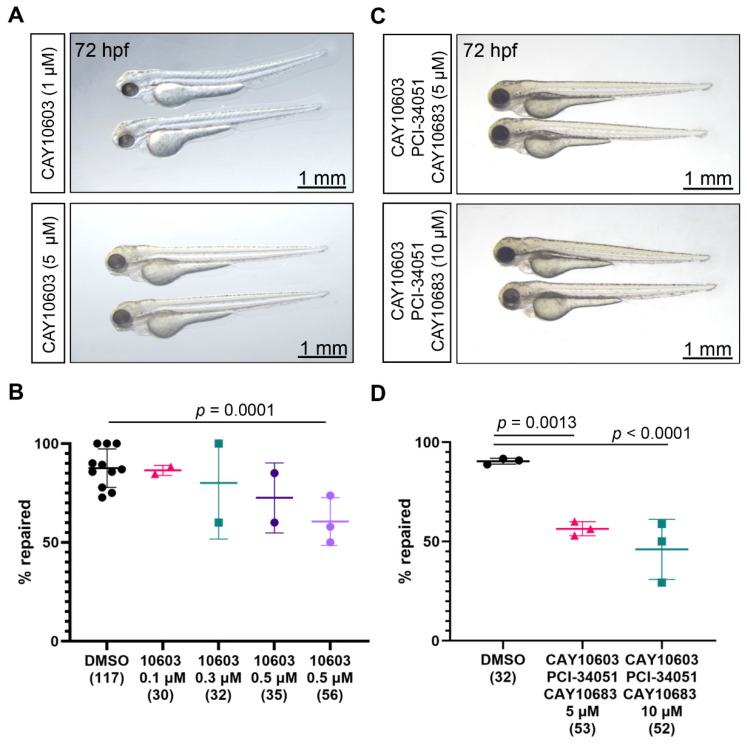
Hdac6 activity is required for zebrafish pronephros repair after injury. (**A**) Development was not affected in embryos incubated in 1 µM (upper panel) or 5 µM (lower panel) solution of the Hdac6 inhibitor CAY10603. (**B**) Only 60% of the embryos treated with 1 µM solution of the Hdac6 inhibitor CAY10603 (10603) repaired the gap after laser-induced injury. The group size is shown in brackets (Fisher’s exact test, two-tailed). (**C**) The combined Hdac2, Hdac6, and Hdac8 activities are required for the migratory response after injury. Incubation of 48 h old zebrafish in solution containing CAY10603, PCI-34051 and CAY10683 (5 µM, upper panel; 10 µM, lower panel) did not affect embryonic development. (**D**) Less than half of the embryos incubated in solution containing CAY10603, PCI-34051, and CAY10683 repaired the tubule after injury. The group sizes are shown in brackets below each group. Individual experiments with mean and standard deviation for each group are displayed. *p*-values were calculated with Fisher’s exact test (two-tailed).

Class IIb consists of HDAC6 and HDAC10, which both have second putative catalytic domain that is not found in other HDAC [23]. Recently, a number of reports have confirmed that pharmacological inhibition of Hdac6 has a protective effect in murine AKI models [18,20,21,25,26]. We used the selective Hdac6 inhibitor CAY10603 (IC50 of 2 pM), to study the function of Hdac6 in the migratory repair response after laser-induced injury of the zebrafish pronephric tubule. Two-day-old embryos, incubated in 1 µM or 5 µM solution of CAY10603, developed normally (Figure 3A). Importantly, CAY10603 treatment reduced the fraction of embryos that successfully repaired in a concentration-dependent manner (Figure 3B). Thus, Hdac6 inhibition negatively affects the early repair after injury of the pronephros.

### 2.4. Combined Inhibition of Hdac2, Hdac6, and Hdac8

Treatment with the broad Hdac inhibitor TSA had a profound effect on the pronephros repair process: only one out of five embryos repaired at 0.4 µM TSA (Figure 1C). In contrast, the pharmacological inhibition of Hdac2, Hdac6, or Hdac8 reduced the fraction of repaired tubuli to a lesser extent. One possible explanation is that downstream targets of Hdac2, Hdac6, or Hdac8 are cumulatively responsible for the stronger TSA effect. To test this possibility, we simultaneously inhibited Hdac2, Hdac6, or Hdac8 in the pronephros ablation model. Treatment of 2-day-old embryos with 5 µM or 10 µM of CAY10603, PCI-34051 and CAY10683 did not interfere with the normal development (Figure 3C). We observed a strong, concentration-dependent effect on the repair process in those embryos; in 10 µM solution, around 2 out of 10 embryos did not repair (Figure 3D). Thus, it is likely that additional TSA targets are involved in the pronephros repair.

### 2.5. Hdac6 and Hdac8 Are Necessary for the Pronephros Repair

**Figure 4:**
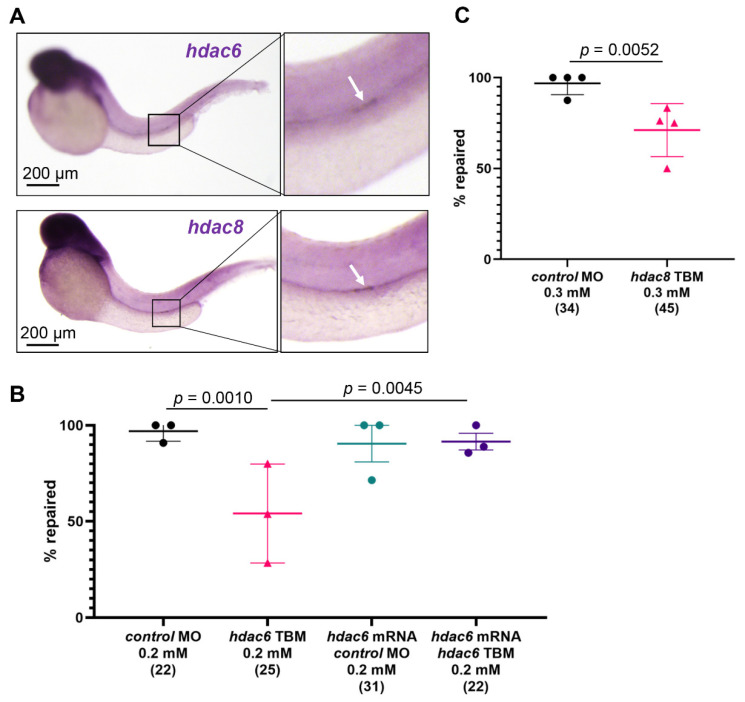
*hdac6* and *hdac8* are necessary for the pronephros repair process. (**A**) In situ hybridization revealed an upregulation of *hdac6* (upper panel) and *hdac8* (lower panel) two hours after laser-induced injury. The arrow points to the injury site. (**B**) Depletion of zebrafish *hdac6* by translation- (TBM) blocking morpholino oligonucleotides (MO) significantly reduced the percentage of embryos that repaired the pronephric tubule (Fisher’s exact test, two-tailed). Co-injection of *hdac6* mRNA rescued the *hdac6* TBM pronephros repair phenotype. (**C**) Depletion of *hdac8* with translation-blocking morpholino oligonucleotides significantly reduced number of repaired pronephri. The group size is shown in brackets. Individual experiments with mean and standard deviation for each group are displayed. Significance was calculated with Fisher’s exact test (two-tailed).

We have previously shown that genes involved in the repair process are upregulated in the epithelium flanking the injury [7,8]. Whole-mount in situ hybridization revealed increased expression of *hdac6* and *hdac8* in response to injury (Figure 4A). To assess whether *hdac6* and *hdac8* levels affect the repair response, we depleted *hdac6* and *hdac8* using previously characterized translation blocking (TBM) morpholino oligonucleotides (MO) [41,42]. Depletion of either *hdac6* (TBM, 0.2 mM), or *hdac8* (TBM, 0.3 mM) significantly increased the number of embryos that did not repair (Figure 4B,C). Furthermore, co-injection of *hdac6* mRNA and *hdac6* TBM rescued the pronephros repair phenotype, confirming the specificity of the *hdac6* knockdown (Figure 4B). These results further support our findings that *hdac6* and *hdac8* play a role in the migratory response after injury.

### 2.6. Depletion of Hdac6 Suppresses the Migratory Response

**Figure 5:**
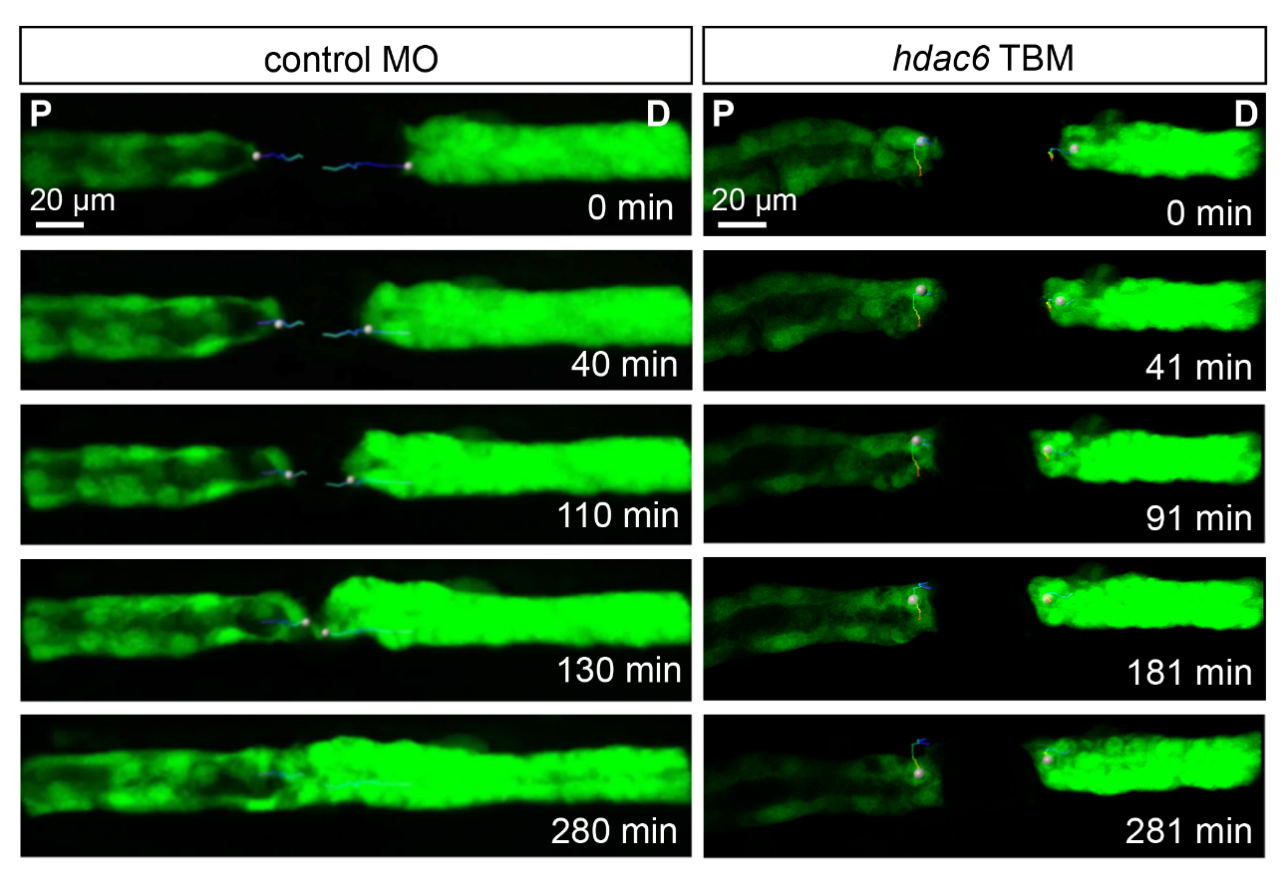
*hdac6* depletion affects the migratory response after laser-induced injury. Frames from a time-lapse movies of control MO-injected and *hdac6* TBM-injected embryos after an injury. While the control embryos closed the 80 µm gap within three hours, the cells flanking the injury in hdac6 TBM-injected embryos showed little to no migratory response (P: proximal; D: distal). The trajectories of one proximal and one distal leading cell per sample are depicted with color-coded lines (blue: early times; red: late times).

Given the profound effect of *hdac6* depletion on the outcome of laser-induced injury of the zebrafish pronephros, we examined the migratory behavior of the injury-flanking epithelial cells. High-resolution time-lapse video microscopy of the repairing pronephros revealed a rapid migratory response in the control group (*control* MO, 0.4 mM); the 80 µm gap was bridged in around 2 h, and the patency of the duct was re-established by 4.5 hpi (Figure 5, Movies S1 and S2). The fast cell migration was accompanied by extensive lamellipodia and filopodia formation in the leading cells (Movies S1 and S2). In contrast, the injury flanking epithelium in *hdac6*-depleted embryos (*hdac6* TBM, 0.4 mM) remained mostly motionless with strongly reduced cellular activity. The size of the gap remained unchanged over 4.5 h of observation (Figure 5. Our data indicate Hdac6 is an essential component of the migratory response after pronephros injury.

### 2.7. Cooperation between NF-κB Signaling and Histone Deacetylase Activity Promotes the Repair Process

**Figure 6:**
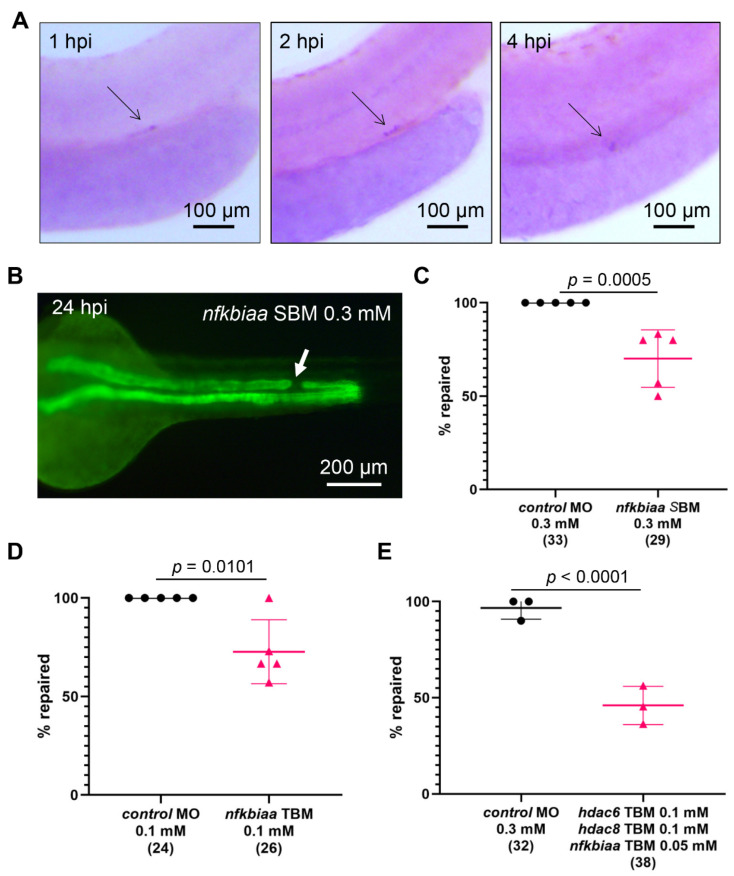
Histone deacetylases and NF-κB signaling cooperate to promote repair through migration. (**A**) Whole-mount in situ hybridization reveals that *nfkbiaa* is upregulated in the cells flanking the injury site and the expression is persistent in the first 4 h post injury (hpi). (**B**) An example of *nfkbiaa* SBM-injected embryo 24 h post ablation. The white arrow points to the injury site. (**C**) Depletion of *nfkbiaa* with splice blocking MO (SBM) significantly reduced the number of successful repairs. (**D**) *nfkbiaa* TBM-injected embryos repair at significantly lower rate than controls. (**E**) Combined depletion of *hdac6* TBM, *hdac8* TBM, and *nfkbiaa* TBM with low MO concentrations synergistically reduces the number of embryos that successfully repair the pronephros. The number of examined embryos is shown in brackets. The points represent individual experiments. Mean and standard deviation for each group are displayed. Significance was calculated with Fisher’s exact test (two-tailed).

Acetylation/expression correlation analysis using enrichment profiles of 130 transcription factor binding sites included in the JASPAR database indicated that TSA may preferentially target promoters with binding sites for NF-κB [43]. Furthermore, HDAC inhibitors interfere with the NF-κB-dependent transcription, and prevent recruitment of RNA polymerase II at target promoters [44,45]. Given the involvement of the NF-κB pathway in cell migration and metastasis [46,47], we hypothesized that histone deacetylases may cooperate with NF-κB to support the migratory response of the zebrafish tubular epithelial cells after injury. Using WISH, we found that the zebrafish *nfkbiaa* is upregulated in the repairing epithelium 1 h after injury, and the *nfkbiaa* expression was persistent for up to 4 hpi (Figure 6A). To examine its role in the migratory response, we depleted *nfkbiaa* with splice blocking MO (*nfkbiaa* SBM) and with previously characterized translation blocking MO (*nfkbiaa* TBM, [48]). Embryos, injected with 0.1mM *nfkbiaa* TBM or 0.3 mM *nfkbiaa* SBM developed normally with a light body curvature (Figure S6). Since the *nfkbiaa* SBM binds to and blocks the splice donor site at the exon 3/intron 3 boundary, we utilized RT-PCR to determine the SBM efficiency. *nfkbiaa* SBM caused intron retention, which leads to a frame shift and a stop codon shortly after (Figure S7). Importantly, depletion of *nfkbiaa* with SBM or TBM resulted in a significant decrease in the number of successful repair events after pronephros injury (Figure 6B–D). Thus, NF-κB signaling is important for the tubular repair. To test for cooperativity between histone deacetylases and the NF-κB pathway, we combined low concentrations of *hdac6* TBM (0.1 mM), *hdac8* TBM (0.1 mM), and *nfkbiaa* TBM (0.05 mM), which did not impair the zebrafish development (Figure S8). We observed a significant delay in the repair process in the triple MO-injected embryos in comparison to controls (*control* MO, 2.5 mM; Figure 6E). Thus, histone deacetylase activity and NF-κB signaling cooperate to promote the immediate migratory response in zebrafish pronephros repair.

## 3. Discussion

Recently, there has been substantial interest in the application of HDAC inhibitors to ameliorate AKI, since many of those inhibitors are either in clinical trials, or have already been approved in oncology treatments [49,50,51]. Suppression of HDAC activity in murine models of AKI has been shown to attenuate the pathological phenotypes. Arguably, best studied is the role of HDAC6 in AKI. In mouse models of AKI, HDAC6 was upregulated after injury, and blocking HDAC6 activity appears to ameliorate the renal pathology [18,20,21,25,26]. Tubostation A (TA), a specific HDAC6 inhibitor, significantly improved renal function, reduced serum creatinine and blood urea levels, and decreased NGAL expression in rhabdomyolysis-induced AKI [20]. This was accompanied by diminished apoptosis in the kidney epithelium, reduced the phosphorylation of NF-κB, the expression of inflammatory cytokines, and the macrophage infiltration [20]. Similar effects were observed in a mouse model of cisplatin-induced AKI [18]. In addition, HDAC6 inhibition with TA attenuated renal pathological changes, reduced the expression of Kim1, tumor necrosis factor-α (TNF-α) and interleukin-6 (IL-6) [18]. Other inhibitors of HDAC6, 23BB and F7, also improved the renal function in the cisplatin and the rhabdomyolysis models by reducing apoptosis, decreasing ER stress, and generally suppressing the inflammatory response [21,26]. Notably, in the mammalian models the data are recorded days after the tubular injury, and HDAC inhibition is most likely necessary to inhibit the transition from AKI to chronic kidney disease (CKD) [52].

In the zebrafish pronephros model, we find that Hdac2, Hdac6, and Hdac8 inhibition strongly suppressed the migratory response during repair. Similar to the murine model, Hdac6 was upregulated after injury. The flanking epithelial cells in Hdac6-depleted embryos exhibited reduced cellular dynamics, and formed fewer lamellipodia and filopodia than controls. Furthermore, the percentage of embryos that successfully repaired the injury was significantly reduced after pharmacological inhibition or triple MO-mediated depletion of Hdac2 Hdac6, and Hdac8. These differences to the murine model probably due to the differences in the windows of observation: the repair response via migration is immediate and takes place within minutes after injury. Suppressed migratory behavior upon HDAC inhibition has been previously described in various disease models, particularly in cancers [49,50,53,54,55]. For example, in human head and neck squamous cell carcinoma (HNSCC) and in colorectal cancer, TSA treatment inhibited cancer cell migration and invasiveness [53,54]. Interestingly, the antitumor effect of TSA was associated with dynamic rearrangement of F-actin in HeLa cells, a process required for cell migration [56]. Thus, it is likely that the upregulation of HDAC6 is beneficial at the early stages of repair to promote cellular behavior, but it becomes contraindicative at later stages, when inflammation and fibrosis drive the transition to CKD.

In rodent AKI models, the NF-κB pathway is activated, stimulates the transcription of cytokines and antiapoptotic genes, and finally leads to inflammation [57]. However, the NF-κB signaling is also known to play an important role in cell migration and metastasis [46,47]. We have previously shown that the directed cell migration after injury depends on the Cxcl12a–Cxcr4b axis [7]. In turn, migration to CXCL12 requires IKKα and IKKβ-dependent NF-κB signaling [58,59]. We found that the zebrafish *nfkbiaa* is not only upregulated shortly after injury, but also that *nfkbiaa* depletion with MO significantly reduced the success rate of pronephros repair. Furthermore, combining *hdac6*, *hdac8*, and *nfkbiaa* MO at low concentrations synergistically delayed the repair process, indicating the joint activities of histone deacetylases and NF-κB are required for the directed cell migration. This is not surprising, since NF-κB-dependent transcription is known to be sensitive to HDAC inhibition [44,45], and TSA shows increased affinity to NF-κB-occupied promoters [43]. Thus, the NF-κB pathway appears to play different, but important roles depending on the timing and the biological context, turning it into a “double-edged sword”. In the very early times after injury, NF-κB promotes the tubular cell migration, which might be important to repair rapidly the damaged epithelium. It is possible that localized cues from damaged cells, such as ATP and ATP metabolites, might act upstream of the NF-κB signaling pathway in the flanking epithelium [8,60]. However, at later time points NF-κB is one of the major signaling pathways activated through the Toll-like receptors (TLR) and it regulates the expression of numerous pro-inflammatory cytokines and chemokines, thereby driving inflammation [61,62]. Since suppression of inflammation via targeting the NF-κB pathway has proven to ameliorate AKI, the timing of inhibition might be relevant in developing treatment protocols.

Our results could guide future studies on therapeutic intervention in cases, when patients are admitted to the hospital shortly after the accident. One example is rhabdomyolysis, where AKI is the most common systemic complication [63,64]. 7–10% of all AKI cases are a complication of rhabdomyolysis [63]. Common drivers of rhabdomyolysis-associated AKI include myoglobinuria, volume depletion, and metabolic acidosis, which are managed at early stages of disease progression [64]. However, rodent models of rhabdomyolysis are well established and could be used to investigate, whether a therapeutic time window exists, when activation of HDAC activity or the NF-κB pathway might stimulate the repair process and avoid loss of kidney function.

Our approach is not without its limitations. While the zebrafish pronephros model enables us to visualize and study the very early repair, it is limited in its ability to model a complete metanephric response to AKI. In order to find their way into translational research, our findings need to be validated in rodent models of AKI, which more closely resemble the human disease. In the complex metanephric kidney multiple cell types are involved in the post-injury events and the immune response plays an essential role in disease progression. We also used only 48 h old embryos in our studies. Analysis at different time points could provide new insights. For example, 24 h old embryos cannot repair through migration [7]. It would be interesting to see whether HDAC or NF-κB pathway stimulation could overwrite the developmental programs and initiate the repair process at earlier stages.

With these caveats in mind, our study shows that the cooperative action of NF-κB and Hdac are required for the immediate migratory response in the zebrafish model of AKI. Further studies in rodent models that utilize timed inhibition of HDAC and the NF-κB pathway could be beneficial for the development of supportive treatment protocols.

## 4. Materials and Methods

### 4.1. Zebrafish Lines Maintenance and Pharmacological Treatment

Zebrafish lines were raised and kept as previously described [65]. Laser ablation studies were performed in the *Tg(wt1b:GFP); Tg(cdh17:GFP)* double transgenic line [7]. Stock solutions of CAY10603 (Merck, Darmstadt, Germany), PCI-34051 (Merck, Darmstadt, Germany), CAY10683 (Biomol, Hamburg, Germany), GSK-J4 (Merck, Darmstadt, Germany), CPI-169 (Biomol, Hamburg, Germany), EPZ5676 (Biozol, Eching, Germany), RN-1 (Merck, Darmstadt, Germany), MS-275 (R&D Systems, Minneapolis, USA), and trichostatin A (TSA, Merck, Darmstadt, Germany) were prepared in DMSO. All working solutions were diluted in Danieau’s buffer to a final concentration of 0.5% DMSO. Control embryos were incubated in Danieau’s solution with 0.5% DMSO. Embryos were incubated in the pharmacological inhibitors 1 h before cell ablation and for 24 h post cell ablation. All animal work was carried out in accordance with the relevant national guidelines (Regierungspräsidium Freiburg).

### 4.2. Photon Laser-Induced Injury, Iepair Quantification, and Image Acquisition

A total of 80 µm of thee pronephric tubule of 2-day-old zebrafish larvae were ablated as previously described [7,8]. Cell ablations were performed with a 2-photon laser (Chameleon) connected to an LSM 880 Observer confocal microscope (Carl Zeiss, Jena, Germany). The repair status was monitored and quantified 24 hpi on a Leica MZ16 epifluorescent stereo microscope (Leica, Solms, Germany). For time-lapse movies, confocal Z-stacks were recorded every 10 min with a C-Apochromat 40×/1.2 objective (Carl Zeiss, Jena, Germany) on the LSM 880 microscope. Z-stacks of the injury site were recorded every 10 min. Three-dimensional reconstruction and time-lapse movie export was carried out in Imaris (Bitplane, Zürich, Switzerland).

### 4.3. Whole-Mount In Situ Hybridization (WISH)

Whole-mount in situ hybridization (WISH) was performed as previously described [66]. Zebrafish embryos were fixed in PFA over night at 4 °C and transferred in methanol for long term storage. cDNA library from 1–2-day-old embryos were used to generate RNA probes against *hdac6* and *hdac8* using gene specific primers for PCR amplification and pCRII-Topo vector (Invitrogen, Carlsbad, CA, USA) for cloning. Sp6 or T7 RNA (Roche, Mannheim, Germany) were used to transcribe anti-sense RNAs from the linearized TOPO vectors. The following primers were used to generate the gene specific fragments for WISH:
*nfkbiaa*-IS-F5′-GACAATATGCGAGCCTTGGG–3′*nfkbiaa*-IS-R5′–GATCCAGGTTCTGCAGGTCT–3′*hdac8* fwd15′-TCTGCCCATTCAATTTCACA–3′*hdac8* rev15′-GAAGAAGCGCCACATGTTTT–3′*hdac6* fwd15′–TTCCCAAACTCAGAGGATGC–3′*hdac6* rev15′–TGGTCTAGAGAAGGCGGAGA–3′


### 4.4. Morpholino Oligonucleotides (MO)-Mediated Gene Depletion and RT-PCR

MO were obtained from Gene Tools, Philomath, USA. *p53*-MO was coinjected in all cases to reduce the unspecific effects [67]. 4 nl of MO diluted in 100 mM KCl, 0.1% phenol red and 10 mM HEPES (pH 7.5) were injected in zebrafish embryos at the 1-cell stage. Injected embryos were kept at 28 °C in Danieau’s solution until further analysis. For mRNA rescue experiments, the *hdac6* open reading frame was amplified from a cDNA library from 2-day-old embryos with gene specific primers. The PCR product was cloned in pCSII vector. *hdac6* mRNA was prepared using an SP6 mRNA polymerase. The following MO were used in this study:
*hdac6* TBM5′-CTTTGGTATCTGGAACCGCATCCAT-3′ [41];*hdac8* TBM5′-ATTACTGTCGCTTTTTTCACTCATT-3′ [42];*nfkbiaa* TBM5′-TGCGGCTCTGTGTAAATCCATGTTC-3′ [48];*nfkbiaa* SBM5′-CTTTCAGATGTGACTGAACTCACCG-3′ (this study);


The *nfkbiaa* SBM targeted the exon 3/intron 3 (e3i3) boundary. The following primers were used for RT-PCR of *nfkbiaa* and *ef1a*:
*nfkbiaa* e3i3 F5′-CCTTGCCATCATTCACGAGG–3′*nfkbiaa* e3i3 R5′-CTTTGCGTCTACATCTGCCC–3′*ef1a* F5′-ATCTACAAATGCGGTGGAAT–3′*ef1a* R5′-ATACCAGCCTCAAACTCACC–3′


## 5. Conclusions

To gain insight into the tubular repair processes after cell ablation of the zebrafish pronephros, we examined the role of histone methylases, histone demethylases, and histone deacetylases using pharmacological inhibitors. Our approach revealed that histone deacetylases are involved in the pronephros repair. Histone deacetylase activity, in particular, Hdac2, Hdac6, and Hdac8, was required for the immediate migratory response, and Hdac inhibition resulted in strongly reduced cellular dynamics. Furthermore, simultaneous depletion of *hdac6*, *hdac8*, and the zebrafish *nfkbiaa* synergistically inhibited cell migration, suggesting cooperativity between HDAC activity and NF-κB signaling in the immediate repair response. Given the reported beneficial effect of Hdac inhibitors in murine models of AKI, our findings imply that the timing of HDAC inhibition might be important for the development of supportive treatment protocols of the human disease.

## Data Availability

Not applicable.

## Supplementary Materials

The supporting information can be downloaded at: https://www.mdpi.com/article/10.3390/ijms23179582/s1.
