# Supplementary material for: Histone Deacetylases Cooperate with NF-κB to Support the Immediate Migratory Response after Zebrafish Pronephros Injury
Source: Int J Mol Sci. 2022 Aug 24;23(17):9582. doi: 10.3390/ijms23179582 (PMC9455417; doi:10.3390/ijms23179582)
Supplement: Supplementary file 1 [file ijms-23-09582-s001.zip › Supplemental_Figures_AKI_HDAC.pdf]

Histone deacetylases cooperate with NF-κB to support the immediate migratory response after zebrafish pronephros injury

Mingyue Zhuang [1], Alexander Scholz [1], Gerd Walz [1,2] and Toma Antonov Yakulov [1,*]

[1] Renal Division, University Freiburg Medical Center, Faculty of Medicine, University of Freiburg, Hugstetter Strasse 55, 79106 Freiburg, Germany
[2] Signaling Research Centres BIOSS and CIBSS, University of Freiburg, Albertstrasse 19, 79104 Freiburg, Germany
* Correspondence: toma.antonov.yakulov@uniklinik-freiburg.de

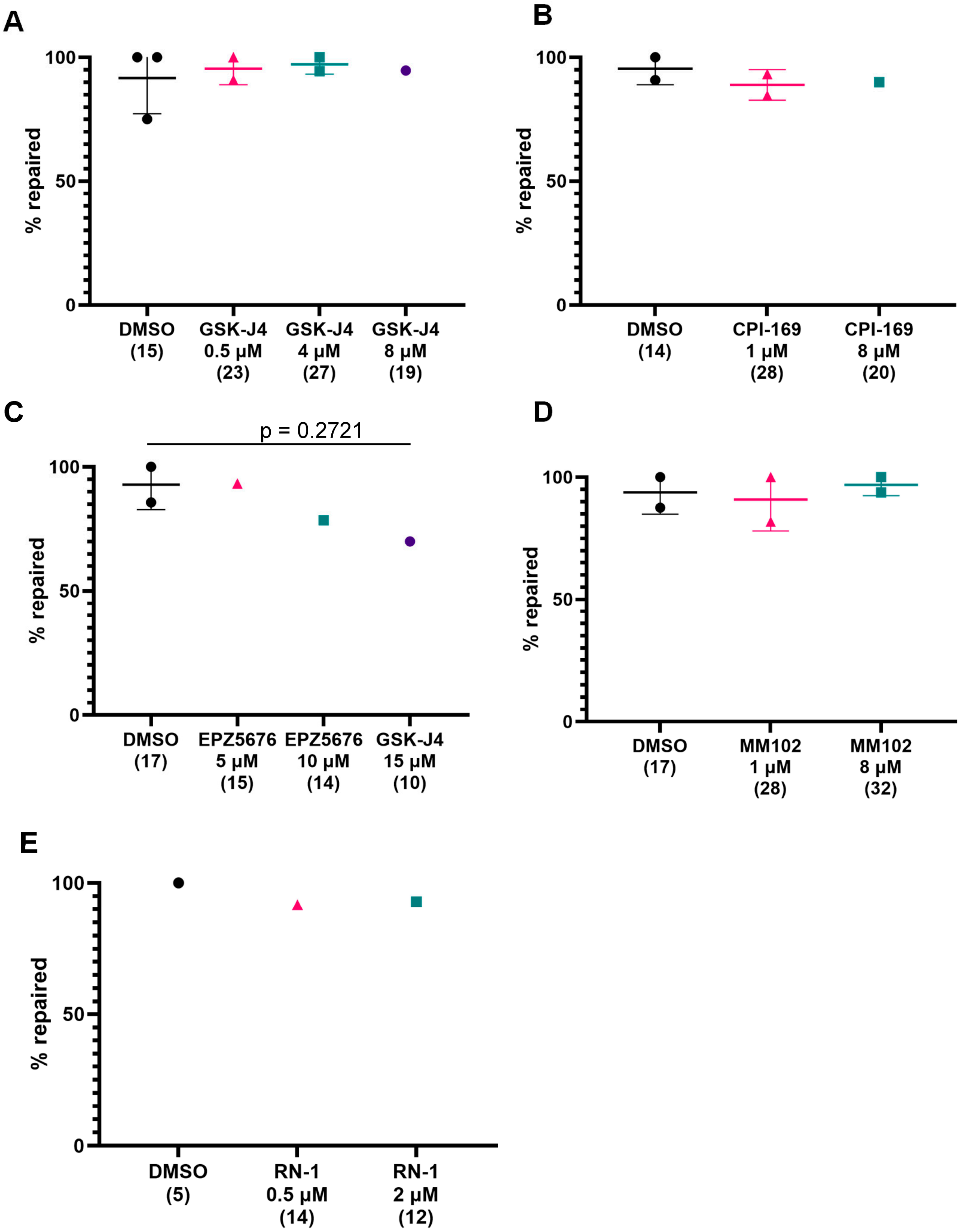

Figure S1. Effect of epigenetic modifiers inhibition on pronephros repair after injury. (A) Treatment with GSK-J4, a histone lysine demethylase 6A/6B dual inhibitor, had no effect on pronephric tubule repair after laser-induced injury in two-day-old zebrafish embryos. (B) Embryos, treated with the Ezh2 inhibitor CPI-169 repaired normally. (C) The Dot1L inhibitor EPZ5676 (Pinometostat) had no effect on the pronephros repair process. (D) Treatment of zebrafish embryos with the Mll1 inhibitor MM-102 did not impede the pronephros repair after injury. (E) Zebrafish embryos, treated with the Lsd1 inhibitor RN1 repaired normally. The group size is shown in brackets. The points represent individual experiments. Mean and standard deviation for each group are displayed. Significance in (C) was calculated using Fisher's exact test, two-tailed.

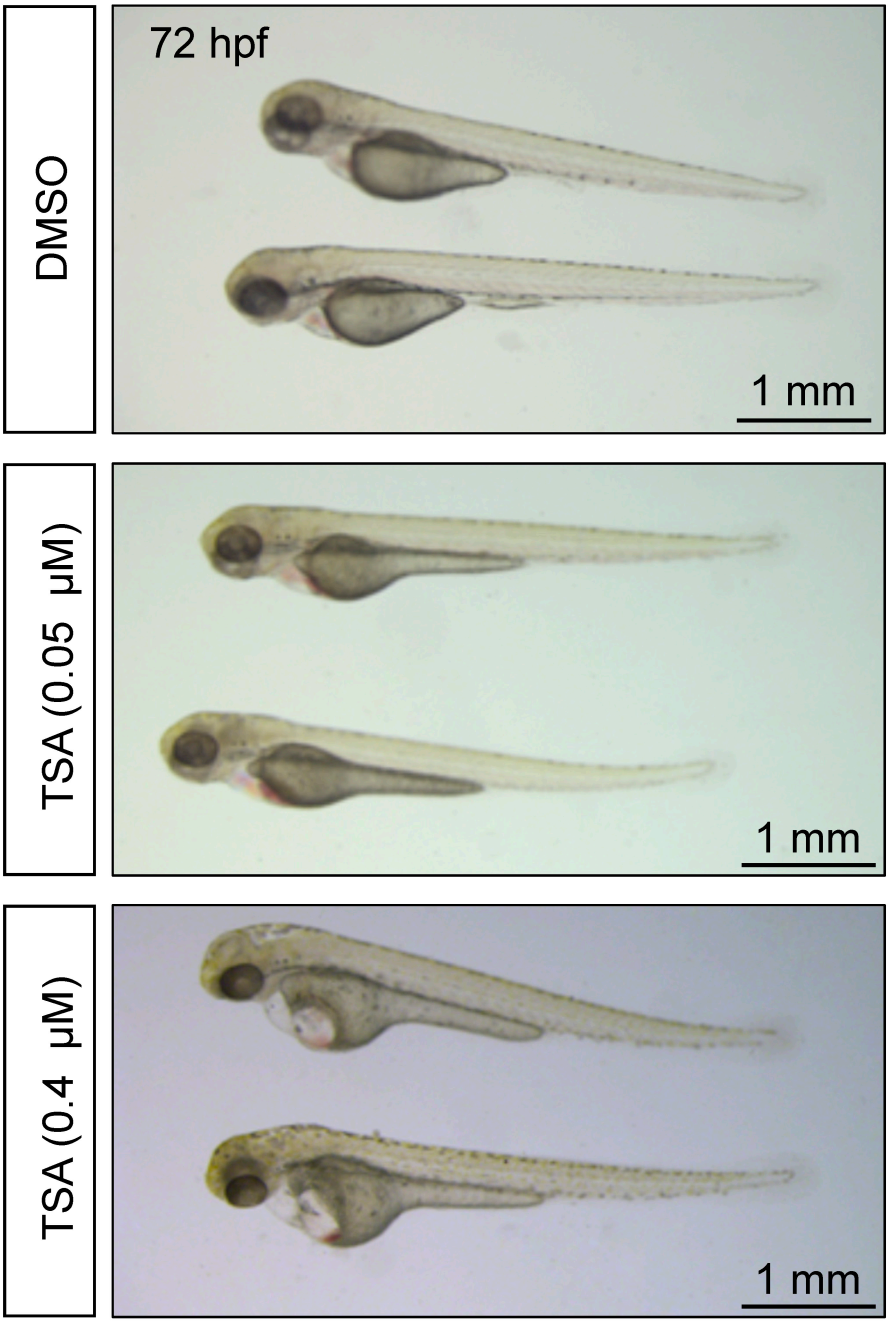

Figure S2. Treatment of zebrafish embryos from 48 to 72 hpf with the pan-Hdac inhibitor TSA. Treatment with 0.05 μM TSA did not affect development (middle panel). Embryos treated with 0.4 μM TSA solution developed slight heart edema.

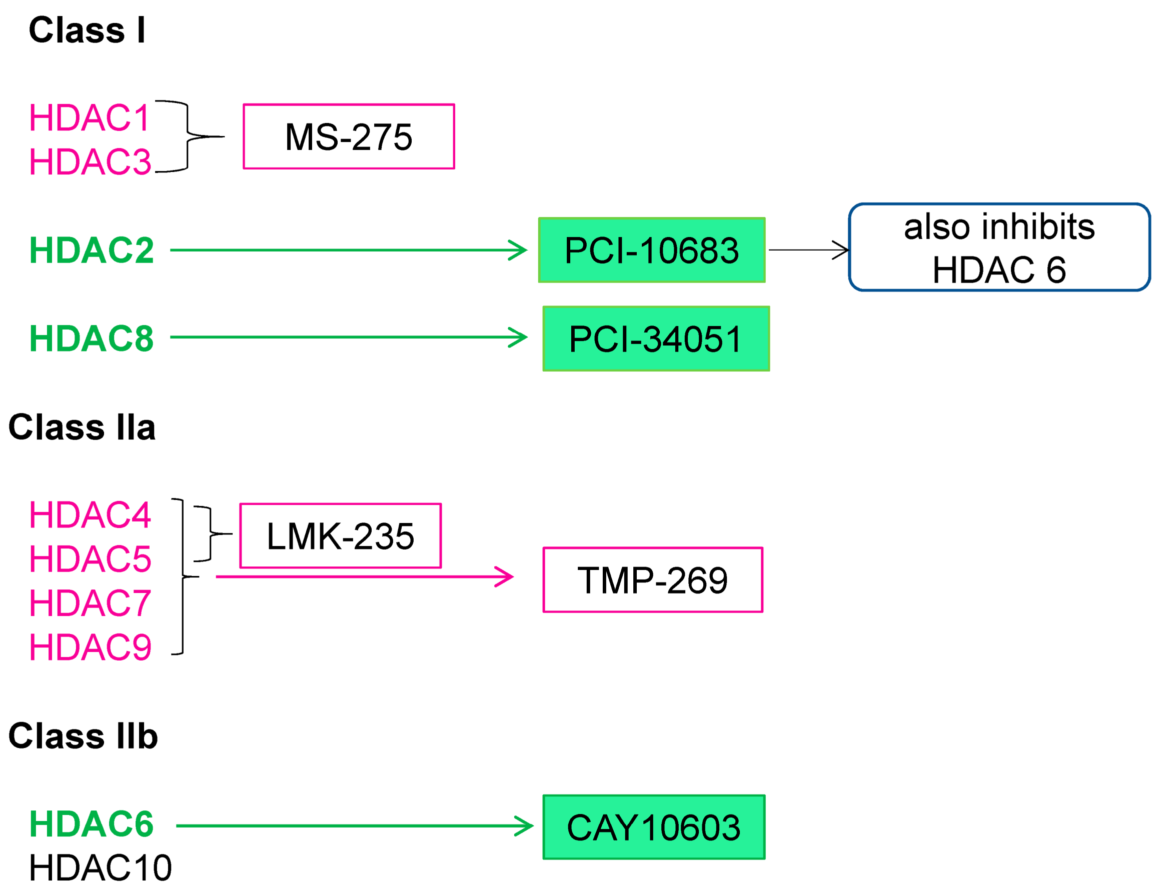

Figure S3. Summary of the histone deacetylase inhibitors used in this study.

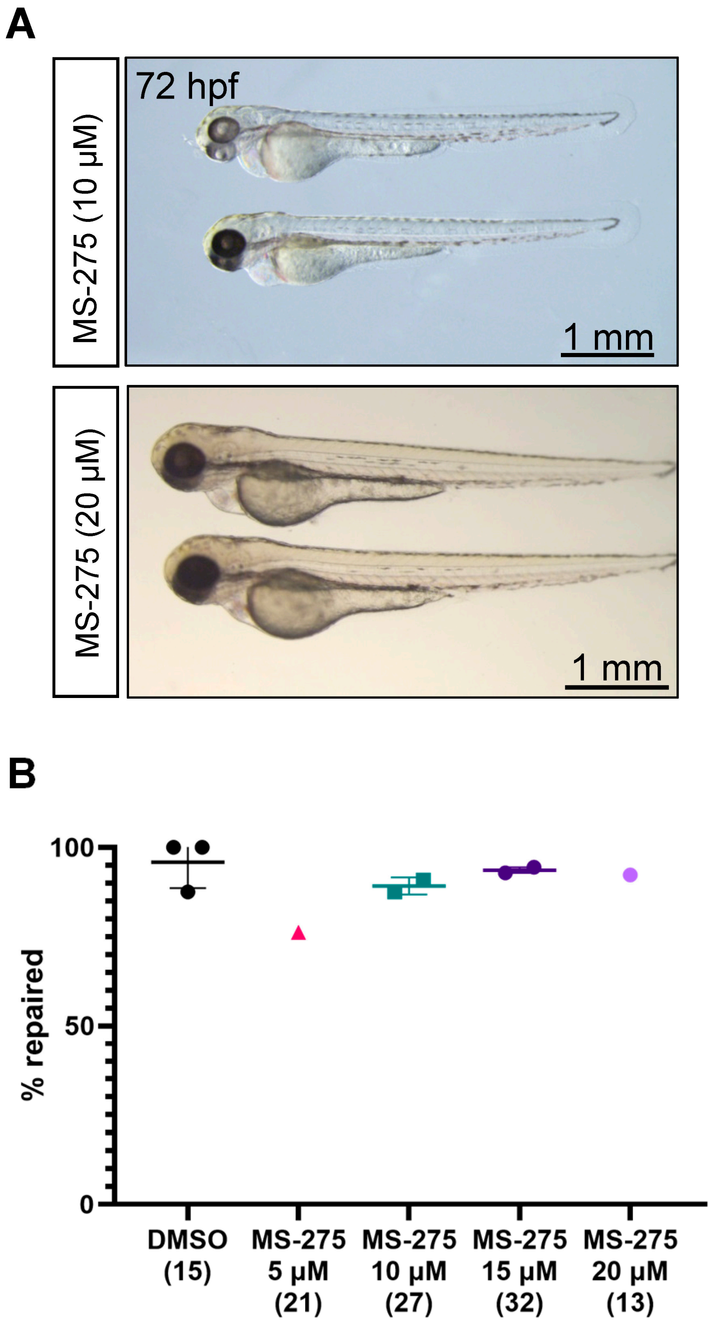

Figure S4. Inhibition of Hdac1/Hdac3 has no effect on the pronephros repair. (A) Treatment of zebrafish embryos with MS-275 did not affect zebrafish development. (B) MS-275 had no effect on pronephros repair after injury. The group size is shown in brackets. The points represent individual experiments. Mean and standard deviation for each group are displayed.

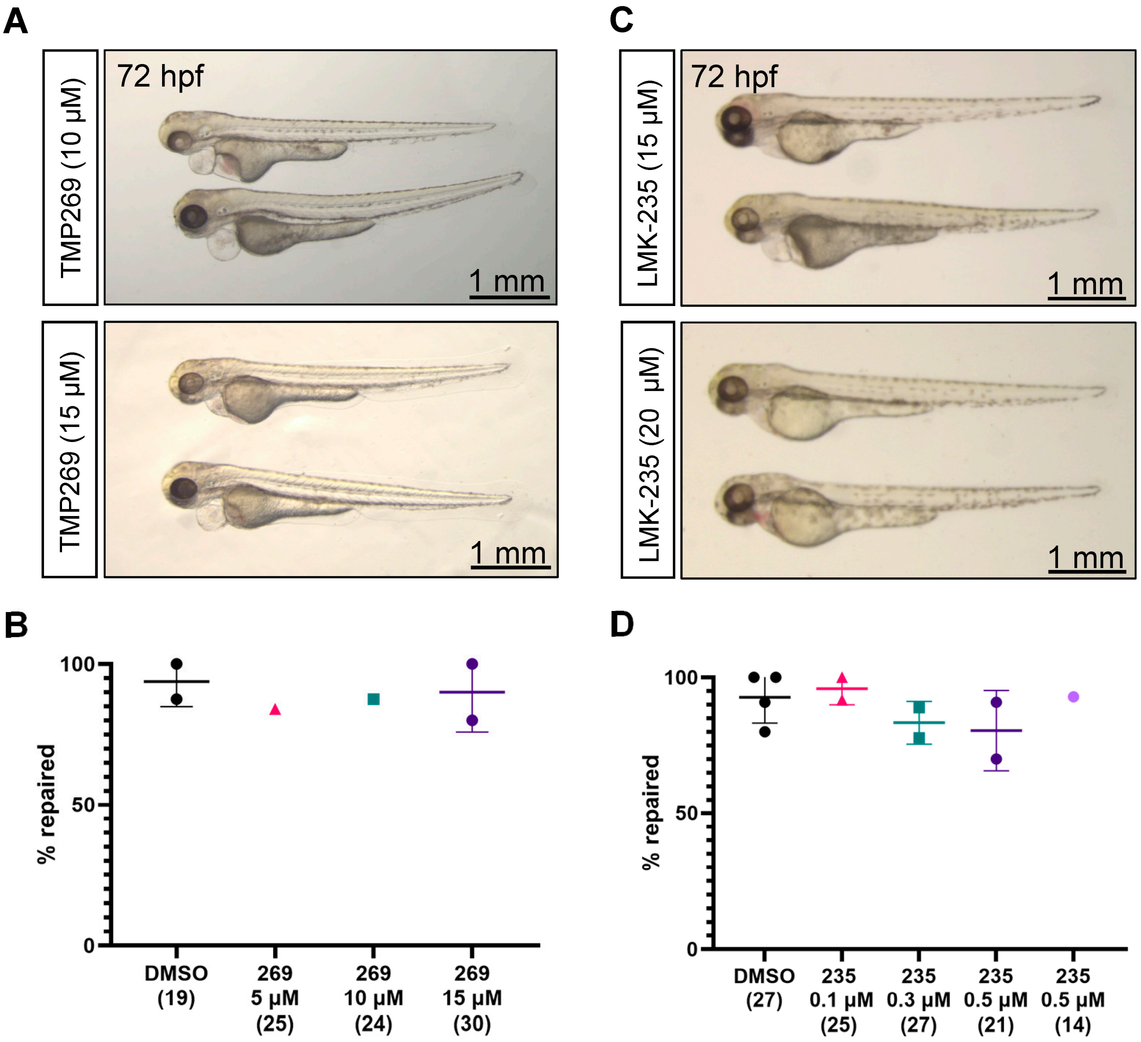

Figure S5. Effect of class IIa Hdac inhibition on pronephros repair. (A) Embryos, treated with the class IIa Hdac inhibitor TMP-269 developed normally. (B) Zebrafish embryos, incubated for 24 h after pronephric injury in TMP-269 repaired normally. (C) Incubation of zebrafish embryos in the Hdac4/ Hdac5 inhibitor LMK-235 between 48 and 72 hpf did not affect development. (D) LMK-235 treatment had no effect on the pronephros repair after laser-induced injury. The group size is shown in brackets. The points represent individual experiments. Mean and standard deviation for each group are displayed.

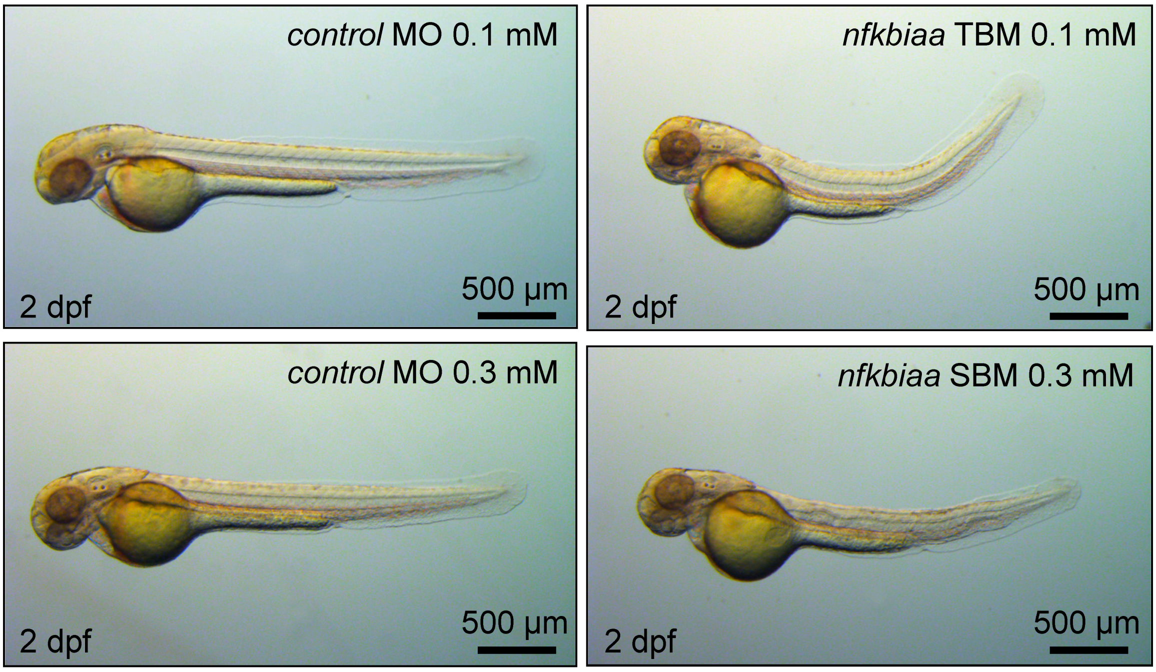

Figure S6. *nfkbiaa* MO-injected embryos develop slight body curvature.

A

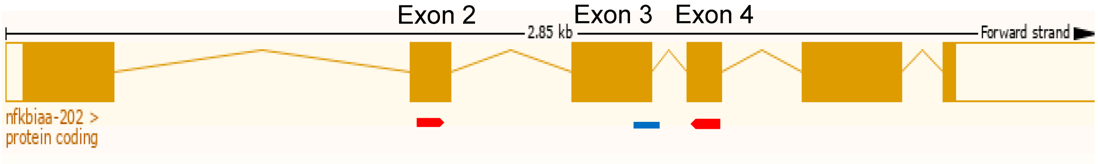

B

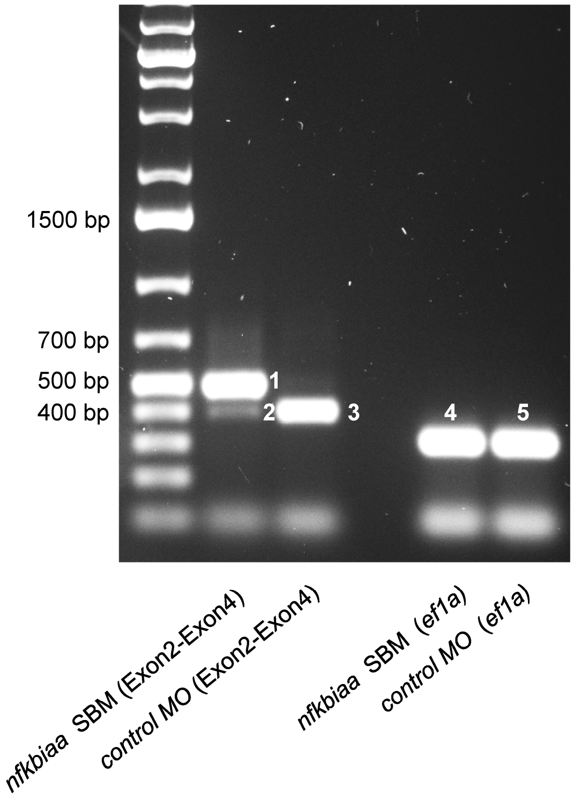

Figure S7. *nfkbiaa* SBM causes intron inclusion. (A) Schematics of the nfkbiaa-202 prodein coding transcript ENSDART00000130107.4 (source https://www.ensembl.org/Danio_rerio/Transcript/Summary?db=core;g=ENSDARG00000005481;r=20:16881883-16884737;t=ENSDART00000130107; accessed 25.11.2021) and the location of the RT-PCR primers (red) in exon 2 (forward) and exon 4 (reverse), as well as the location of the splice blocking MO at the exon3-intron 3 boundary. (B) Semi-quantitative RT-PCR for *nfkbiaa* (Exon2-Exon4) fragment reveals intron 3 inclusion (95 bp) in *nfkbiaa* SBM-injected embryos (major band 1), but not in *control* MO injected embryos. Bands 2 and 3 correspond to the spliced, wild-type sequence. Bands 4 and 5 are control amplifications for the housekeeping *ef1a* transcript.

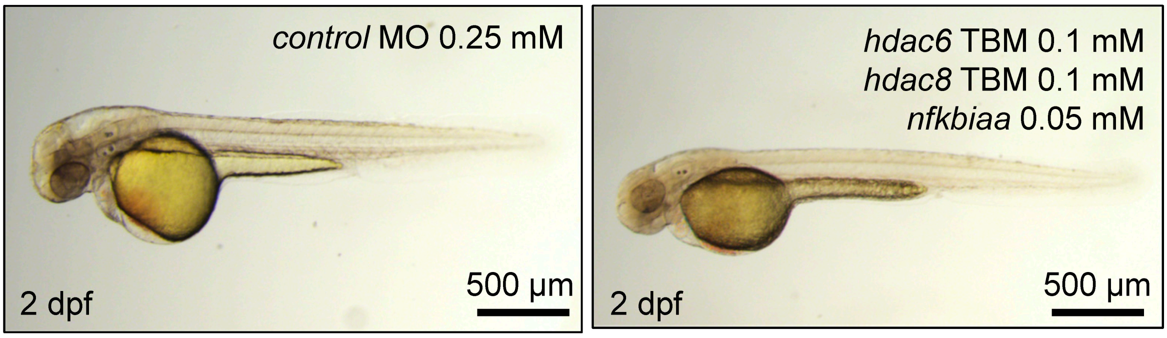

Figure S8. Triple *hdac6 TBM 0.1 mM, hdac8 TBM 0.1 mM, and nfkbiaa 0.05 mM* MO-injected embryos develop normally.

Movie S1. Time-lapse video microscopy of *ctrl*-MO-injected (0.4 mM) zebrafish embryo after laser cell ablation at 48 hpf. Movie S2. Time-lapse video microscopy of *hdac6* TBM-injected (0.4 mM) zebrafish embryo after laser cell ablation at 48 hpf.
